# Cluster2Former: Semisupervised Clustering Transformers for Video Instance Segmentation

**DOI:** 10.3390/s24030997

**Published:** 2024-02-03

**Authors:** Áron Fóthi, Adrián Szlatincsán, Ellák Somfai

**Affiliations:** 1 Department of Artificial Intelligence, ELTE Eötvös Loránd University, 1053 Budapest, Hungary; ek7r2g@inf.elte.hu (A.S.); somfaiellak@inf.elte.hu (E.S.); 2 HUN-REN Wigner Research Centre for Physics, 1121 Budapest, Hungary

**Keywords:** transformers, video processing, instance segmentation, semisupervised learning

## Abstract

A novel approach for video instance segmentation is presented using semisupervised learning. Our Cluster2Former model leverages scribble-based annotations for training, significantly reducing the need for comprehensive pixel-level masks. We augment a video instance segmenter, for example, the Mask2Former architecture, with similarity-based constraint loss to handle partial annotations efficiently. We demonstrate that despite using lightweight annotations (using only 0.5% of the annotated pixels), Cluster2Former achieves competitive performance on standard benchmarks. The approach offers a cost-effective and computationally efficient solution for video instance segmentation, especially in scenarios with limited annotation resources.

## 1. Introduction

Video instance segmentation (VIS) involves identifying and delineating individual objects in a video sequence, while distinguishing between different instances of the same object category. This task combines three main components: object detection: identifying and localizing objects within each video frame; object tracking: keeping track of the same objects as they move across frames, maintaining their identities; and instance segmentation: providing a pixel-level mask for each object instance.

Video instance segmentation plays a key role across diverse applications, such as autonomous driving, surveillance, robotics, and video analysis. By identifying and tracking individual instances of objects, this technique is instrumental in enabling systems to comprehend the details of object movements and interactions within a temporal context in a given video stream. Its applications extend beyond visual comprehension, providing insights into the spatial and temporal relationships among objects. In autonomous driving scenarios, for instance, video instance segmentation is indispensable for real-time decision making, ensuring the safe navigation of vehicles through complex environments. In surveillance and robotics, this technology facilitates the precise monitoring and manipulation of objects, improving the efficiency and accuracy of these systems. Furthermore, in video analysis, the capability to discern and isolate specific instances greatly contributes to extracting meaningful information and patterns from large datasets. As technological advancements continue, the importance of video instance segmentation becomes increasingly apparent, changing the way we perceive and interact with visual data across various domains.

Cutting-edge VIS models currently rely on comprehensive video annotations from VIS datasets [1,2] to face this demanding challenge. However, video annotation is expensive, especially when creating precise object masks. In addition, there are instances where it becomes difficult to precisely delineate the boundaries of objects due to occlusion [3] or blurring. Even the less precise polygon-based mask annotations are significantly more time-consuming than annotating videos with bounding boxes [4,5] or scribbles. This issue is particularly critical for recent transformer-based VIS models [6,7,8], as they have a high demand for training data.

Our objective in this paper is to develop a competitive video instance segmentation model using lightweight annotations. We found that scribbles—free-hand curvy lines drawn across the characteristic parts of the instance—are not only an effective way to provide partial mask information, but also sufficient to achieve competitive performance on VIS benchmarks.

**Figure 1:**
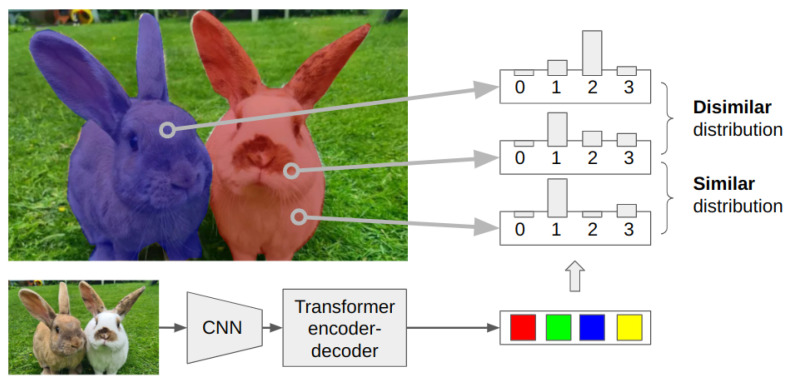
Illustration of the clustering-based approach. Based on the learned object queries, we compute the output of the decoder for a pixel, representative of the features of the object the pixel is embedded in, as a probability distribution. Our learning objective guides the decoder to output similar distributions for pixels embedded in the same object and different distributions for a pixel pair embedded in different objects.

We combine the Mask2Former [7,9] model (to achieve high VIS performance) with similarity-based constraint loss (to enable the semisupervised nature of clustering-based methods). For an illustration of clustering-based methods, see Figure 1.

To our knowledge, our Cluster2Former model is the first VIS method trained in partial masking that achieves high-quality segmentation results (for a survey of recent VIS models, see [10]). The main contributions of this paper are summarized as follows:

- We propose a VIS model, which is trained by scribbles drawn on the training video frames. Our model achieves competitive performance despite using only 0.5% of the pixel count of the full training masks as annotation.
- The above result is achieved by modifying the learning objective only, leaving the architecture of the transformer (in this work, Mask2Former) intact. This not only eliminates costly architecture-specific hyperparameter optimization, but also enables the application of the same loss function modification to future, more advanced VIS architectures.
- We demonstrate that the pairwise approach for training, based on feature vectors obtained by transformers, provides an efficient solution to video instance segmentation.

## 2. Related Works

With the development of the DETR model [11], a paradigm shift took place in the field of image instance detector development. Beforehand, a technical change implemented to improve performance typically complicated the algorithm to such a degree that further incremental enhancements became increasingly difficult. Starting with DETR, it has been important to maintain the simple and transparent end-to-end scheme, replacing proxy-based approaches. This has enabled developments such as the Mask2Former model [9] for still images, which, due to its simplicity, reaches a state-of-the-art level in solving several different tasks, such as semantic segmentation, instance segmentation, and panoptic segmentation. With a minor change, Mask2Former became capable of handling video instance segmentation, beating the competition at the time of its introduction [7].

Clustering-based approaches like [12,13] have already shown that they can achieve state-of-the-art performance in proposal-free instance segmentation. They utilized one important property of instance labeling: the pairwise relationship between pixels as the supervision to formulate the learning objective. That leads to a semisupervised clustering problem, which we also employ. This approach beats the conventional two-stage method (feature embedding with k-means) by a significant margin [12].

In a related work, pairwise constraints and subset allocation have been employed to redefine similarity-based constraints and accurately utilize strong-supervised information [14]. The efficacy of pixelwise clustering techniques has been demonstrated in challenging video instance segmentation tasks, such as tracking identical objects, utilizing pairwise clustering methods [15].

CMT-DeepLab [16] has a targeted segmentation as a pixel clustering problem. It considers the object queries as cluster centers, and is responsible for grouping pixels for segmentation. That way, it improved the performance of the existing approaches, and achieved a state-of-the-art result on COCO test-dev. Although it achieved a remarkable result, the price of this was the complexity of the architecture, which limits its applicability in various research areas.

Clustering, in particular incorporating clustering features into the learning process, has been shown to improve image segmentation with class imbalanced datasets [17]. The precision of the segmentation at critical edges can be enhanced by using a supervised edge attention module [18].

We aim to keep the idea of the cluster-based approach and provide a much simpler solution, which can then be further successfully developed to target other areas, like video instance segmentation and multiview problems.

Video instance segmentation (VIS) stands as a significant domain within computer vision, tasked with the challenging goal of simultaneously detecting, tracking, and segmenting objects in video sequences. Unlike conventional image-based tasks, VIS operates in the dynamic realm of videos, necessitating the ability to identify objects across multiple frames and provide precise pixel-level segmentation masks for each object instance. To gain a better understanding of this field, it is important to categorize the existing methods as two-stage approaches and one-stage approaches. The two-stage approaches [1,19,20] first tackle object detection in each frame, and then proceed to perform instance segmentation. It is akin to the well-established two-stage architecture seen in image-based tasks and includes well-known models like faster R-CNN [21] and mask R-CNN [22]. One-stage methods, on the contrary, integrate object detection and instance segmentation into a single process. Although they are more computationally efficient, they may sacrifice accuracy. YOLACT [23] and BlendMask [24] are examples of one-stage approaches.

Track-then-segment approaches [25,26] initially focus on object tracking across video frames, and subsequently apply instance segmentation. They rely on specialized tracking algorithms to establish object identities across frames before segmentation. Recent advances in deep learning have led to end-to-end deep learning models [27] that directly address video instance segmentation, often using temporal information to enhance results. Online and real-time methods [28,29,30,31] are tailored for applications like autonomous vehicles and robotics; these methods are optimized for real-time or online video processing, emphasizing low-latency inference. Multiobject tracking and segmentation [32,33,34,35,36,37] aim to track and segment multiple objects simultaneously to address complex scenarios involving multiple interacting or overlapping objects.

Temporal consistency models [6,16,27,28,31,38,39] tackle object tracking and segmentation challenges by leveraging temporal relationships between frames in videos. Attention-based models, which use attention mechanisms to focus on relevant frame details at different time steps, excel in capturing object motion, occlusion, and appearance changes over time. This makes attention a crucial component for maintaining consistency in video instance segmentation. Most of these models use full pixel-level mask annotation of the objects to be segmented. In contrast, our approach only uses a fraction of this during training. CMT [16] is a transformer-based segmentation framework that transforms traditional transformer architectures for segmentation and detection to utilize object queries as cluster centers, which play a pivotal role in pixel grouping for segmentation. The clustering process involves two alternating steps: initially assigning pixels to clusters based on feature similarity and subsequently updating cluster centers and pixel features. Cluster2Former follows similar principles, but only with the application of the training objective, without changing the architecture.

Given the expense and complexity of annotating videos, semisupervised and weakly supervised methods aim to reduce annotation requirements by using fewer annotated frames or less detailed annotations (such as bounding boxes) for training. In the early stages of VIS, there were explorations of using videos for segmentation tasks that involve weak, semisupervised, or unsupervised methods, with a focus on motion or temporal consistency. However, many of these earlier methods did not specifically tackle object coherence and relied on optical flow for frame-to-frame matching. An approach to unsupervised feature learning leverages low-level motion-based grouping cues [40], resulting in an effective visual representation trained using unsupervised motion-based segmentation on videos. Ref. [41] predicts segmentation masks of multiple instances by learning instance tracking networks using labeled images and unlabeled video sequences. MinVIS [42] achieves VIS performance without specialized video architectures by training an image-based instance segmentation model and treating video frames as independent images, thanks to its query-based approach for temporal consistency and memory-efficient inference online. MaskFreeVIS [5] achieves competitive VIS performance using only bounding-box annotations. The approach leverages temporal mask consistency through the temporal KNN-patch loss without any labeled masks, significantly reducing annotation costs. That method outperforms the optical flow-based baselines, using bounding box annotation. While boundary boxes require even less information than the scribble method we employ, it can be misleading in scenarios of significant occlusion, making uncertain which object it encompasses. Additionally, for objects with ambiguous boundaries, obtaining precise human annotation becomes challenging.

## 3. Methods

In this section, we present an overview of the key aspects of our proposed methodology. We explain how scribble-based annotation can effectively represent and replace the full mask ground truth, describe the loss function that can capture data from partial annotation, provide details on our strategy to select positive and negative pairs, and finally show how all this is put together and trained.

### 3.1. Annotation Based on Scribbles

As mentioned in the Introduction, in this paper, we advocate the utilization of partial annotation in the form of scribbles. Scribbles are continuous curvy lines passing through representative parts of the image of the instance.

**Figure 2:**
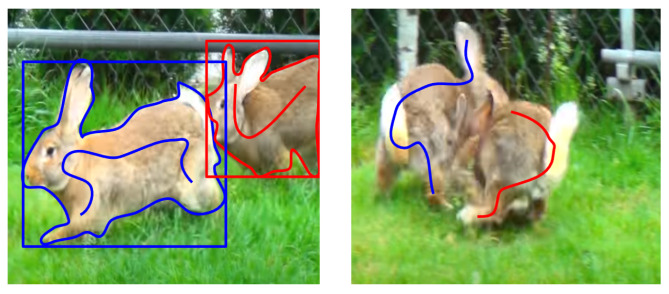
Annotations—the first instance is marked by blue, the second by red. (**Left**): Three annotation methods are displayed: full mask (shown as a surrounding boundary), bounding box (minimal enclosing rectangle), and scribble. (**Right**): For a more challenging example containing both occlusion and blurry regions, neither the full mask nor the bounding box can provide unambiguous annotation. Using scribbles, however, one can select representative regions with high confidence.

While the full mask contains complete information about the pixels of the instance, partial annotations such as a bounding box and scribbles (see Figure 2 (left) for illustration) have their own advantages. Scribbles have the edge, especially for more challenging situations, where the annotator can select regions of high confidence, avoiding ambiguous locations; see Figure 2 (right) for illustration.

We return to a detailed comparison of the different annotation techniques in the Section 5.

### 3.2. Similarity-Based Constraint Loss

A novel learning objective was introduced in Ref. [13] to train deep neural networks to perform end-to-end image pixel clustering, with a specific focus on instance segmentation. This method takes advantage of the fundamental pairwise relationships between pixels in the context of instance labeling as a form of supervision. It utilizes the Kullback–Leibler (KL) loss to encourage the model to generate distinct representations for pixels belonging to different objects while producing similar representations for pixels associated with similar objects. The resulting clusters serve as direct instance labeling, contributing to the field of image segmentation.

Meta-classification learning (MCL) is a multiclass classification technique that relies on pairwise similarity information instead of class-specific labels. The method optimizes a binary classifier for predicting pairwise similarities, ultimately learning a multiclass classifier as a submodule. MCL provides several notable advantages for various machine learning and computer vision applications. First, it simplifies the model training process by eliminating the need for hyperparameter tuning, resulting in more straightforward and efficient development. Second, MCL excels in complex and dynamic scenarios, demonstrating robustness in handling diverse data types and variations in data distribution. Its flexibility allows it to adapt to the nuances of challenging settings. Additionally, MCL is well suited for unsupervised and semisupervised learning, making it valuable in situations with limited labeled data. ClusterRCNN [15] has exploited these properties to track identical individuals. Through pairwise clustering of pixels on consecutive frames, we adapt it for to the requirements of VIS.

The similarity-based constraint loss is a key element for guiding our semisupervised clustering process. We employ the pairwise constraint and subset allocation deep embedded clustering (PCSA-DEC) approach [14], as it has shown the effectiveness of similarity-based constraint loss over other pair-based clustering procedures. The method relies on the comparison of point pairs, distinguishing between “positive” pairs (where the points from the pair belong to the same cluster) and “negative” pairs (where the points from the pair belong to different clusters). A cluster can be considered the region of an object or the background, and a point is represented by a feature vector *p*, whose components are the results of the queries with a softmax applied. The positive and negative point–point relationships are represented by a binary vector *R*, where the entries are set to 1 for a positive point pair and 0 for a negative pair.

For each point pair, we compute the cosine similarity $S^{\cos}$, which ranges between −1 and 1. The cosine similarity quantifies the degree of resemblance between the two points:

$$S_{ij}^{\left(\cos\right)}=\frac{p_{i}\cdot p_{j}}{|p_{i}\left|\;\right|p_{j}|},$$

where $p_{i}$ and $p_{j}$ are the feature vectors of the points *i* and *j*. The cosine similarity is transformed into a dissimilarity measure *D*, in the range [0,1], by

$$D_{ij}=\frac{1-S_{ij}^{\left(\cos\right)}}{2},$$

which is clamped to the interval [ϵ,1−ϵ] to ensure numerical stability for the calculation of the cross-entropy in the next step. We used $\epsilon =10^{-6}$ in our numerical experiments.

The core of the similarity-based constraint loss function involves a cross-entropy loss *L*, designed to minimize the deviation between each pair of points based on the computed dissimilarities *D* and the positive–negative relationships *R*:

$$R_{ij}=\left\{\begin{array}{c} 1\;\mathrm{if}\;i\;\mathrm{and}\;j\;\mathrm{are}\;\mathrm{in}\;\mathrm{the}\;\mathrm{same}\;\mathrm{instance}\\ 0\;\mathrm{otherwise}. \end{array}\right.$$

This loss is computed as

$$L_{ij}=-R_{ij}\log\left(1-D_{ij}\right)-\left(1-R_{ij}\right)\log\left(D_{ij}\right).$$

Additionally, to account for variations in the importance of point pairs based on their distances, a weighting mechanism is introduced; see Section 3.3 for details. The weights *W* are applied to the computed loss, resulting in a weighted loss term. Finally, the similarity-based constraint loss $L_{\mathrm{SC}}$ is obtained as the mean across the *N* point pairs (ij) of the weighted loss terms:

$$L_{\mathrm{SC}}=\frac{1}{N}\sum_{\left(ij\right)}L_{ij}W_{ij}.$$

The total loss for training is then

$$L=\lambda_{\mathrm{cls}}L_{\mathrm{cls}}+\lambda_{\mathrm{SC}}L_{\mathrm{SC}},$$

where $L_{\mathrm{cls}}$ is the classification loss (as in Mask2Former), and $\lambda_{\mathrm{cls}}$ and $\lambda_{\mathrm{SC}}$ are hyperparameters.

### 3.3. Pairing Strategies

The performance of our method on VIS benchmarks critically depends on the way pixel pairs are sampled. In the context of semisupervised clustering, to keep resource requirements under control, we do not use all pixels within the instance mask, unlike the previous approaches [12,13,22].

**Figure 3:**
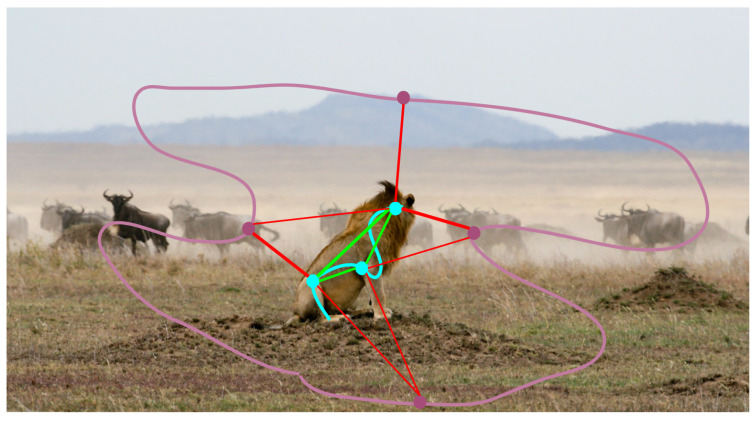
Selection of pixel pairs in foreground and background regions. The foreground object (lion) is annotated by a cyan scribble, the background by a pink scribble. Positive pairs (green lines) are selected between points of the foreground object, but not between points of the background. Negative pairs (red lines) are selected between points of different clusters: in this figure between the background and the foreground object. This way, the model is not forced to recognize the background as a single homogeneous object; instead, it can segment into unlabeled distinct clusters, e.g., other animals, ground, mountain, and sky in this figure.

**Figure 4:**
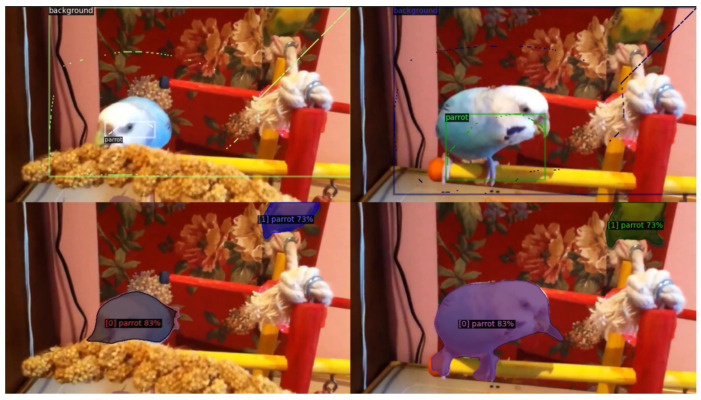
Benefits of omitting the positive pairs between background points. **Top** row: ground truth frames. The scribbles are shown at the original resolution (1 pixel wide curves), and the bounding boxes are displayed only as visual guides for the scribbles. **Bottom** row: prediction. The parrot at the top-right corner of both frames is detected correctly, even though it was not annotated. By not enforcing similarity between different regions of the background, it can maintain its heterogeneity.

Our approach leverages the flexibility and robustness of transformers, mitigating the limitations associated with similarity-based constraint losses used with convolutional networks [12,13]. In particular, we do not employ binary preclassification to separate foreground and background, and we do not impose an upper limit on the number of possible clusters. Instead, our upper limit is determined by the number of transformer queries. Therefore, annotating the background with a simple scribble, similarly to the instances, is sufficient. We make a distinction between how we select positive pairs for individuals and background. While we choose positive pairs within individual instances, we do not do so for the background; see Figure 3 for illustration. This choice permits the model to autonomously segment the background into multiple clusters, a practical solution given that the semantic classes in the training data do not encompass all objects in the video. Consequently, distinct and clearly discernible entities may appear on the unannotated background, and we do not constrain the model to interpret them as a single entity, as demonstrated in Figure 4.

Within the same (foreground) instance, we establish positive connections between all selected pixels, the pairs being weighted by

$$W_{ij}^{\left(\mathrm{positive}\right)}=w_{\min}+\left(1-w_{\min}\right)\frac{d_{ij}}{d_{\max}},$$

where $d_{ij}$ is the Euclidean distance between the points *i* and *j*, $d_{\max}$ is the maximum possible distance (the diagonal of the image frame), and $w_{\min}$ is a hyperparameter. This way, faraway pixels of the same instance, including those separated by occlusion, are helped to be clustered together. Conversely, for negative pixel pairs from different instances, we assign weights decreasing with distance, preventing nearby, similar objects to be grouped as a single entity:

$$W_{ij}^{\left(\mathrm{negative}\right)}=1-\left(1-w_{\min}\right)\frac{d_{ij}}{d_{\max}}.$$

To address the variations in object sizes and the availability of annotated pixels, we adjust the sampling to select roughly equivalent portions from each object. This not only enhances the training process, but also reduces the use of computational and memory resources. Furthermore, we introduce sparsification into our approach (removing some of the low-weight negative pairs), recognizing that separating distant, dissimilar objects is straightforward. Therefore, retaining only a fraction of the pairs is sufficient to achieve our clustering objectives.

For further implementation details, see Section 4.2.

### 3.4. Architecture and Training

Leveraging the Mask2Former [7] architecture, our approach introduces pivotal changes in the training process, while maintaining the model’s original structure. Inspired by [13], we adopt a similar pixel sampling strategy. This involves randomly sampling points from scribble-based ground truth masks for segmentation. Our method diverges in the use of similarity-based constraint loss for segmentation and cross-entropy loss for classification. The Hungarian matcher is adapted to incorporate this new loss, ensuring that Cluster2Former retains the inference efficiency of the original Mask2Former.

## 4. Results

In this section, we present details about the datasets used, the implementation, and the experimental results. We provide detailed ablation studies in addition to standard benchmark results to illustrate the effectiveness of individual parameter settings and their combinations.

### 4.1. Datasets

We conducted our experiments using the datasets YouTube-VIS 2019 and 2021. The YouTube-VIS 2019 dataset consists of 2883 videos with annotations for 131,000 object instances spanning 40 categories. To address more intricate scenarios, the 2021 version of YouTube-VIS introduces an additional 794 training videos and 129 validation videos, featuring tracklets with intricate motion trajectories.

We made a scribbled version of both datasets. With the DAVIS Interactive Robot [43,44]—which generates realistic scribbles that simulate human interaction—we modified the annotations for the training process. Instead of the original ground truth masks, which cover the whole objects, we used the scribble annotations.

### 4.2. Implementation Details

**Figure 5:**
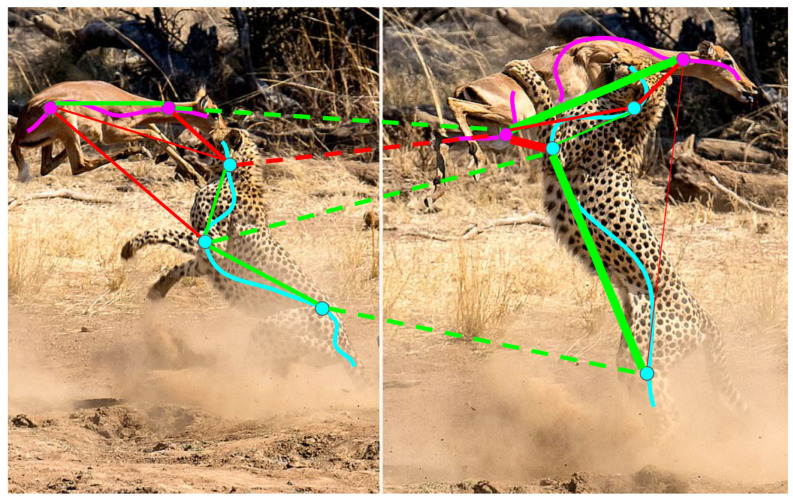
Pairing of points on consecutive frames. The two objects are annotated by a magenta scribble (antelope) and cyan scribble (cheetah). The dots on the scribbles are sampled pixels (for clarity, only a few representative sample pixels and pixel pairs are shown in this figure). The positive pairs are connected by green lines, the negative pairs by red lines. The thickness of the connecting lines represents the weight of the pair: close negative and distant positive pairs are given heavier weight. The lines connecting pixels in different frames are drawn by dashed lines.

As mentioned in the Section 3, we adapt Mask2Former [7] (especially sampling and training loss), but keep the architecture unchanged. Unless otherwise specified, all other training schedules and settings are kept the same as in the original model. To generate the pixel pairs for the similarity-based constraint loss, 300 pixels are sampled randomly from each frame foreground scribble, distributed evenly between the instances on that frame, as well as 300 pixels from the background scribbles. The choice of framewise sample count (as opposed to instancewise sample count as in [13]) enables good memory control. In case either the foreground or the background scribbles contain less than 300 possible pixels for sampling, e.g., due to cropping, the sample count is reduced for both to keep the balance. We make two disjoint sets of pixel-pair connections: in-frame and interframe connections. The in-frame connections contain a pixel pair, where the two pixels come from the same frame, while the interframe connections contain pixel pairs, where the two pixels come from different frames. When only a single frame is considered, there are no interframe connections set. For in-frame connections, we connect each pixel from the instances with all other pixels from the instances (they can be positive or negative pairs, depending on whether the pixels are from the same instance) and with the background pixels (these are all negative pairs). In the case of interframe connections for each frame pair, we connect each instance pixel from one frame with all other instance pixels from the other frame (they can be positive or negative pairs; see Figure 5). Additionally, we connect the background pixels for each of the two frames with all instance pixels from the other frame (negative pairs). We do not use pixel-pair sparsification—we do not drop out any connection. We set the prefactor of the similarity-based constraint loss to $\lambda_{\mathrm{SC}}=10$ (keeping $\lambda_{\mathrm{cls}}=2$) and the minimum weight to $w_{\min}=0.9$. In case of inference, after applying a softmax for the mask logits along the queries dimension, we produce the predictions by thresholding the given values with 0.1. In this way, a point makes a prediction mask if its value is bigger than 0.1. We call this hyperparameter the inference threshold.

Originally, Mask2Former [7]-based models are trained with a batch size of 16 and a learning rate of $10^{-4}$. We adapted these to our hardware resources, which were initially two NVIDIA RTX TITAN GPUs, followed by NVIDIA A100 GPUs. If we do not specify it otherwise, we use a batch size of 4 with a learning rate of $2.5\times 10^{-5}$. We also increased the number of iterations to 24k (32k) and the steps for the learning rate decay to 16k (22k) for the YouTube-VIS 2019 (2021) val datasets to allow the model to see the same number of inputs as with the original configuration. As the architecture is unchanged, the computational requirements for prediction (inference) are identical to those of Mask2Former.

### 4.3. Experiments

Our first results, shown in Table 1, demonstrates the power of our method. We compare the standard YouTube-VIS average precision and average recall benchmarks [1] for different model configurations on the YouTube-VIS 2019 val dataset. The integration of similarity-based constraint loss with Mask2Former’s segmentation losses (first row) showed no change in performance. When training with our Cluster2Former schedule (few sampled pixels only, randomly selected from the full mask; third row), a slight decay is observed, which we attribute to the information reduction of sampled pixels vs. full mask use (note that Mask2Former also samples pixels, but 12,544 of them with the default configurations). Our main point is the fourth row: when the sampling of Cluster2Former is performed from pixels with a scribble curve, only a slight reduction in performance is observed, while the training involves typically 100 times less pixels.

The same experiment has been performed on the more challenging YouTube-VIS 2021 val dataset as well; see Table 2. It is remarkable that simply adding similarity-based constraint loss (second row) outperforms the original Mask2Former model without altering resource requirements. We stress that if, unlike the rest of this paper, the objective is to use full annotation masks to achieve best segmentation performance, then the best strategy is to take the linear combination of Mask2Former’s original mask losses with the similarity-based constraint loss. This dataset is more difficult than the 2019 version, which is reflected in the benchmark figures for model configurations using only the pairwise-sampling-based loss. We offer a possible explanation in Section 5.

Next, we compare our system with a few (original) state-of-the art models for the two datasets; the results are presented in Table 3 and Table 4. Similar conclusions can be drawn as above.

### 4.4. Ablation Experiments

In the context of our ablation study within the YouTube-VIS 2019 validation set and using the ResNet-50 backbone, we meticulously dissect Cluster2Former. As our baseline VIS method, we employ Mask2Former [7], fully integrating it into our approach, the only modification being the replacement of mask losses with our custom losses. We analyze the distinct components of the pixel-pair selection strategies with a focus on the following aspects (see Table 5):

(1) Investigation of the interaction among background pixels. This exploration stems from the hypothesis that allowing our model the freedom to distinguish background elements into multiple clusters could potentially enhance performance.

(2) Examination of pixel relations within instances based on their spatial proximity. By emphasizing distant positive pairs and nearby negative pairs, we aim to facilitate the connection of occluded regions while effectively separating different instances in close contact.

(3) Evaluation of pixel relationships across instances in successive frames, elucidating the impact of temporal relations on tracking. This involves the classification of consecutive pixels belonging to the same instance into a shared cluster.

Furthermore, we extend our analysis to consider scenarios where more than two frames are interconnected in this manner, offering a perspective on the temporal aspect. In this experiment, a varying number of frames (“tube length”) are selected randomly from a 20-frame video sequence; benchmark results are shown in Table 6. As expected, discarding temporal links (tube length 1) deteriorates performance. However, it is interesting to see that the best results are obtained for tube length 2; for a longer tube length, the temporal connections might have been diluted.

## 5. Discussion

In the previous sections, we presented our Cluster2Former model designed to tackle VIS and showed that competitive results can be obtained despite using lightweight, scribble annotation.

Annotation based on scribbles has several benefits compared with both full pixel-level masks and another popular lightweight masks: the bounding boxes. When compared full masks, they have a number of advantages: (1) Scribbles require significantly less annotator time and training. Full mask annotation can be extremely time-consuming and costly, while scribbles are quicker and easier for annotators. Scribbles are more forgiving to annotator errors and require less skills to provide good-quality annotations. (2) Scribbles are less prone to ambiguity: in challenging scenarios with blurred images or a strong object overlap, determining precise object boundaries for full masks can be ambiguous. Scribbles, on the other hand, provide a clear indication of object locations without the need for exact boundary delineation. (3) Scribbles are computationally efficient: training VIS models with scribbles typically requires less computational resources than full mask-based training, making it a more feasible option for resource-limited scenarios.

**Figure 6:**
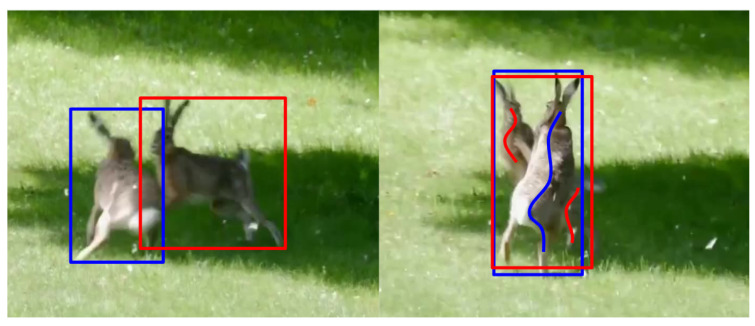
**Left**: Although the image is not completely sharp, the extent of the individuals can be specified with the help of a bounding box. **Right**: The instances are still clearly distinguishable, despite the fact that the edges are blurred, but the bounding boxes that border them almost completely coincide, since the two individuals cross each other. It is not clear which bounding box is the annotation of which instance. On the other hand, the surfaces belonging to each individual can be clearly marked with scribbles. Blue and red bounding boxes and scribbles identify different instances.

It has been shown by MaskFreeVIS [5] that the performance of state-of-the-art traditional VIS algorithms can be approached by using another light annotation: bounding boxes. Still, scribbles offer a number of advantages over bounding boxes as well: (1) Better object separation and reduced ambiguity. Scribbles provide a more effective means of separating objects, especially when they overlap or intersect. The ambiguity of object reference for nearly coinciding bounding boxes is dissolved by applying scribbles in clearly identifiable parts of the image. See Figure 6 for illustration.

(2) Enhanced object location and adaptability to object shape. Scribbles offer finer-grained localization information. Unlike bounding boxes, which encompass a fixed rectangular area, scribbles can guide the model to better capture the object contour, especially complex shapes. (3) Reduced annotation effort. Although both methods are partial annotations, scribbles typically require less annotator time than meticulously aligning bounding boxes to object edges, making the annotation process more efficient.

**Figure 7:**
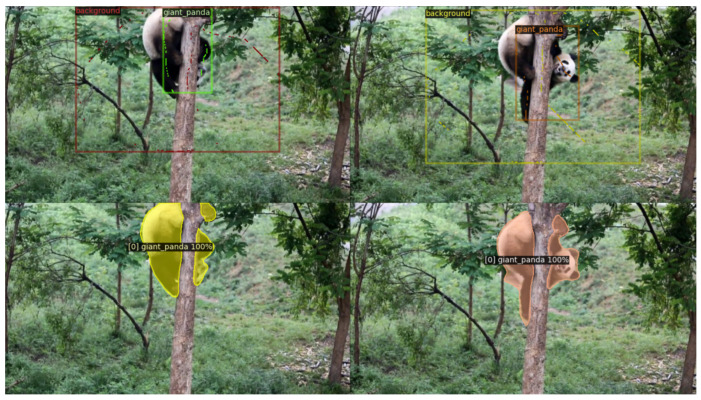
Instance segmentation of an occluded object. **Top** row shows the annotated frames; **bottom** row is prediction. The bounding boxes are only guiding the eye to localize the annotation scribbles, as in Figure 4.

**Figure 8:**
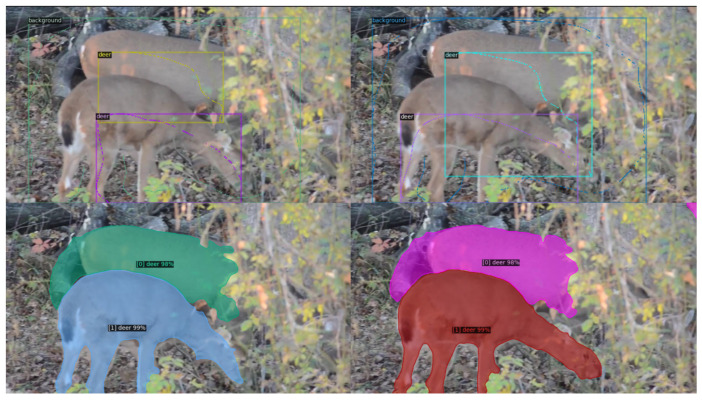
VIS of directly adjacent instances; the separating edge is correctly found.

We show that Cluster2Former can successfully address challenging VIS situations. Figure 7 demonstrates that disjoint parts of an occluded object are combined to make a proper instance. In Figure 8, we show that the edges separating neighboring instances are correctly delineated despite the fact that scribbles provide only an approximation to the full shape.

**Figure 9:**
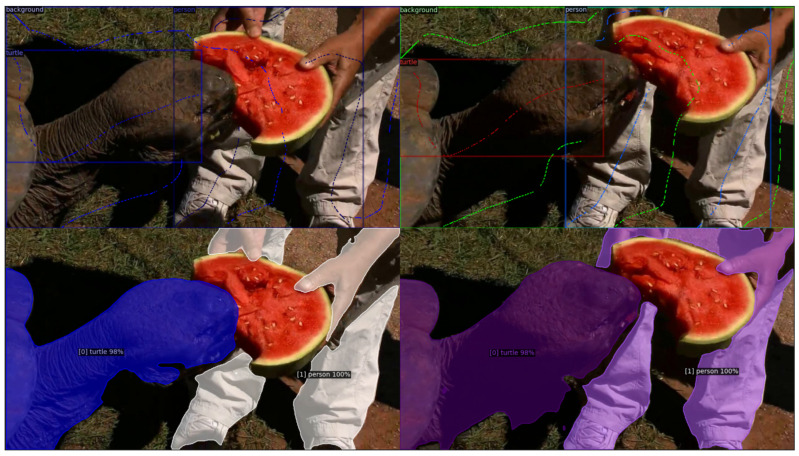
VIS of frames containing many occlusions.

Figure 9 demonstrates that Cluster2Former successfully copes with situations of multiple occlusions. The two objects (turtle and human) are correctly segmented despite the fact that the pixels of the human are split into a number of disjoint regions.

**Figure 10:**
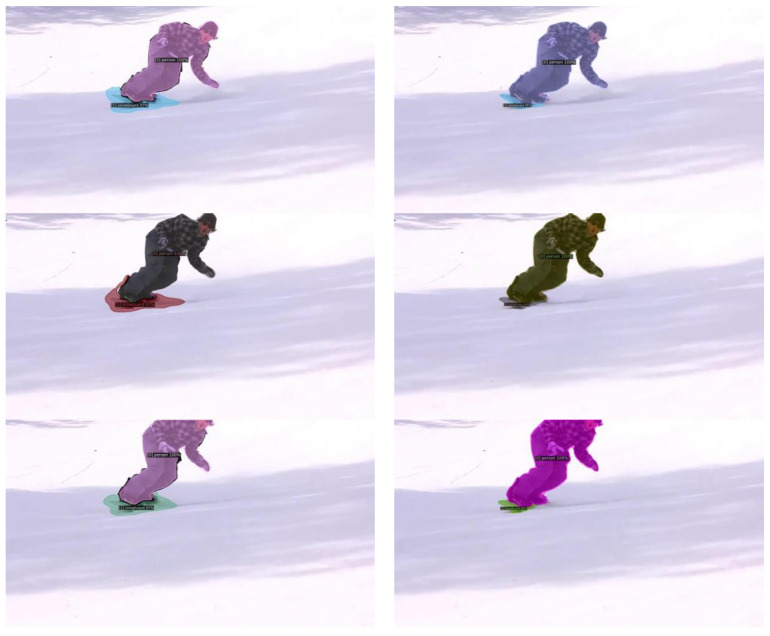
Limitation of our model. **Left** column: frames segmented by Cluster2Former (ours) from the test set of the YouTube-VIS 2021 dataset. Even though both instances (person and snowboard) are correctly found, the detected edge of the snowboard is quite far from the ground truth. **Right** column: the same frames segmented by Mask2Former; the edge of the snowboard is accurate.

Limitations: since the scribbles we use in the experiments (generated by DAVIS Interactive, similar to skeletons) sample pixels typically from the inner parts of the objects, pixels near the edges are less represented; consequently, the segmentation quality near the edges might suffer. See Figure 10 for illustration. On the one hand, this is not necessarily a serious impediment, for example, for applications where tracking of similar instances is the ultimate task. On the other hand, there are solutions to overcome this issue, including using scribbles that approach object edges at places or strengthening the segmentation near the edges, for example, using supervised edge attention [18].

Based on the above arguments, we believe that Cluster2Former can be one of the best overall VIS methods in a number of practical situations when training is limited by annotation resources. Even though, for a fixed-size training dataset, our model is slightly weaker than full mask methods, using a fixed amount of annotator time, much larger training sets can be prepared with scribbles than full masks, resulting in a better overall performance. As an outlook, one of the applications we have in mind is tracking almost-identical-looking individuals (“instances”) of animals, where maintaining the identity of individuals through frames is much more important than segmentation quality. Additionally, we work on an extension of our model, which is capable of handling a mixture of scribbles and full masks. Full or nearly full masks, which are easy for the annotator, enable maintaining the full VIS benchmark of the underlying architecture, but can fall back to scribbles where a full mask is costly or impossible due to blur, still enabling suitable precision for tracking.

## Figures and Tables

**Table 1:** Comparison of different configurations of Mask2Former and our Cluster2Former model on the YouTube-VIS 2019 validation dataset using the ResNet-50 backbone. From top to bottom: the original Mask2Former model, Mask2Former with mask losses and similarity-based constraint loss, Cluster2Former with similarity-based constraint loss using the original full mask annotations, and Cluster2Former with similarity-based constraint loss using the scribble annotations.

Losses	Annotation	AP	AP50	AP75	AR1	AR10
Mask2Former [7]	mask	46.4	68.0	50.0	-	-
Mask2Former [7] + SC loss (ours)	mask	46.3	68.1	50.3	47.7	59.5
Cluster2Former (ours)	mask	41.7	66.1	45.6	42.9	51.5
Cluster2Former (ours)	scribble	38.3	62.5	42.5	39.3	46.6

**Table 2:** Comparison of different configurations of Mask2Former and Cluster2Former on YouTube-VIS 2021 val using the R50 backbone. The model configurations are the same as in Table 1.

Losses	Annotation	AP	AP50	AP75	AR1	AR10
Mask2Former [7]	mask	40.6	60.9	41.8	-	-
Mask2Former [7] + SC loss (ours)	mask	41.6	65.1	44.4	39.0	52.9
Cluster2Former (ours)	mask	34.1	55.8	37.4	33.8	42.3
Cluster2Former (ours)	scribble	29.5	51.7	30.4	30.3	37.2

**Table 3:** Comparison of state-of-the-art models on the YouTube-VIS 2019 val dataset using the R50 backbone.

Method	Annotation	AP	AP50	AP75	AR1	AR10
Mask2Former [7]	mask	46.4	68.0	50.0	-	-
MaskFreeVIS [5]	bbox	43.8	70.7	46.9	41.5	52.3
SOLO-Track [41]	wo video	30.6	50.7	33.5	31.6	37.1
Cluster2Former (ours)	scribble	38.3	62.5	42.5	39.3	46.6

**Table 4:** Comparison of state-of-the-art models on the YouTube-VIS 2021 val dataset using the ResNet-50 backbone.

Method	Annotation	AP	AP50	AP75	AR1	AR10
Mask2Former [7]	mask	40.6	60.9	41.8	-	-
MaskFreeVIS [5]	bbox	37.2	61.9	40.3	35.3	46.1
Cluster2Former (ours)	scribble	29.5	51.7	30.4	30.3	37.2

**Table 5:** Ablation results of Cluster2Former with the ResNet-50 backbone on the YouTube-VIS 2019 val dataset. Checkmarks indicate the application of the following components: “Neg BG Only”: background pixels participate only in negative pairs. “Weighted Pairs”: use weights for the pixel pairs. “Temp Pairs”: temporal pairs employ pairing across consecutive frames.

Neg BG Only	Weighted Pairs	Temp Pairs	AP	AP50	AP75	AR1	AR10
	✓	✓	35.1	59.8	37.5	36.5	43.1
✓	✓		33.8	58.4	36.6	36.2	42.8
✓		✓	36.4	61.2	39.7	39.2	45.4
✓	✓	✓	38.3	62.5	42.5	39.3	46.6

**Table 6:** Results of varying tube lengths (number of frames taking part in temporal pixel pairing) during training on YouTube-VIS 2019 val using the R50 backbone. Tube length 1 denotes the training of the model with only spatial pixel pairs.

Tube Length	AP	AP50	AP75	AR1	AR10
1	33.1	57.9	35.1	35.4	42.4
2	38.3	62.5	42.5	39.3	46.6
3	37.0	61.6	40.8	39.7	46.7
4	35.9	59.6	39.9	38.7	45.5

## Data Availability

Our code is available at https://github.com/szlAdrian/Cluster2Former. The original YouTube-VIS 2019 and 2021 datasets are available publicly. The scribbled annotation of the datasets can be downloaded from https://drive.google.com/drive/folders/17d30cWqAh5Q7czX0yur3Nu0jv9Ez9FHe?usp=sharing (both urls accessed on 26 January 2024).

## Abbreviations

The following abbreviations are used in this manuscript:

MCL	meta-classification learning
PCSA-DEC	pairwise constraint and subset allocation deep embedded clustering [14]
VIS	video instance segmentation
